# A Case of Acute Aortic Dissection Type B Associated with Cushing's Syndrome

**DOI:** 10.4021/jocmr2009.02.1224

**Published:** 2009-03-24

**Authors:** Luigi Petramala, Dario Cotesta, Paolo Sapienza, Laura Zinnamosca, Enrico Moroni, Luca di Marzio, Giorgio De Toma, Claudio Letizia

**Affiliations:** a Day Hospital of Internal Medicine and Hypertension-Department of Clinical Science University "Sapienza", Rome, Italy; b Department of Surgery "P.Valdoni", University "Sapienza", Rome, Italy

## Abstract

We report a case of a 63-year-old man, with a previous history of hypertension and glucose intolerance associated troncular obesity that was emergently admitted to our Institution for evaluation of a severe, constant posterior chest pain which radiated anteriorly and dyspnoea with a suspected diagnosis of acute aortic dissection. A CT scan of thorax and abdomen demonstrated a dissection starting just below left succlavian artery and extending downward to the left renal artery, involving the celiac tripod and superior mesenteric artery. The dissection was classified as Stanford B, De Bakey III. Moreover, CT scan of abdomen revealed incidentally a left adrenal tumor of 25 mm of diameter. An emergent prosthetic graft was placed just below the origin of the left succlavian artery up-to the diaphragmatic hiatus. Furthermore, a diagnostic evaluation of the mass revealed an increase of cortisol production, and a diagnosis of Cushing's syndrome was done and the patient underwent an adrenalectomy via laparotomic approach. We report an association of acute aortic dissection of acute aortic dissection type B associated to Cushing's syndrome.

**Keywords:**

Cushing's syndrome; Adrenocortical adenoma; Aortic dissection type B

## Introduction

Cushing's syndrome (CS) occurs in about 2 per million of the general population [1]. CS refers to the signs and symptoms caused by an excessive action of glucocorticoids [2, 3]. Endogenous CS results from chronic exposure to excess glucocorticoids produced by the adrenal gland. It may be caused by excess ACTH production (80-85%) usually due to a pituitary adenoma. In this form of the disease, a female to male ratio of 8 to 1 is observed, with an average age ranging from 20 to 40 years among affected women [4].

Less frequently it is caused by extrapituitary tumour (ectopic ACTH syndrome) or, very rarely, by tumour secreting CRH. Furthermore CS can also be ACTH independent (15-20%) when it is due to excess secretion of cortisol by unilateral adrenocortical tumours, either benign or malignant, or as consequence of bilateral adrenal hyperplasia, especially in cyclic CS.

CS is frequently associated with cardiovascular risks due to hypercortisolism, leading to cardiovascular complications, including myocardial infarction, congestive heart failure and stroke account for high proportion of morbidity and mortality in untreated patients [5, 6].

Although CS has been known as a risk factor for aorta dissection, the association of these two entities is extremely rare [7, 8]. We report a case of an acute aorta dissection in a patient with CS caused by adrenocortical adenoma.

## Case Report

A 63-year-old man with history of hypertension and glucose intolerance since age 59 years was emergently admitted to our Institution from a peripheral Hospital for evaluation of a severe, constant posterior chest pain, radiating forward, and dyspnoea with a suspected diagnosis of acute aortic dissection.

**Figure 1:**
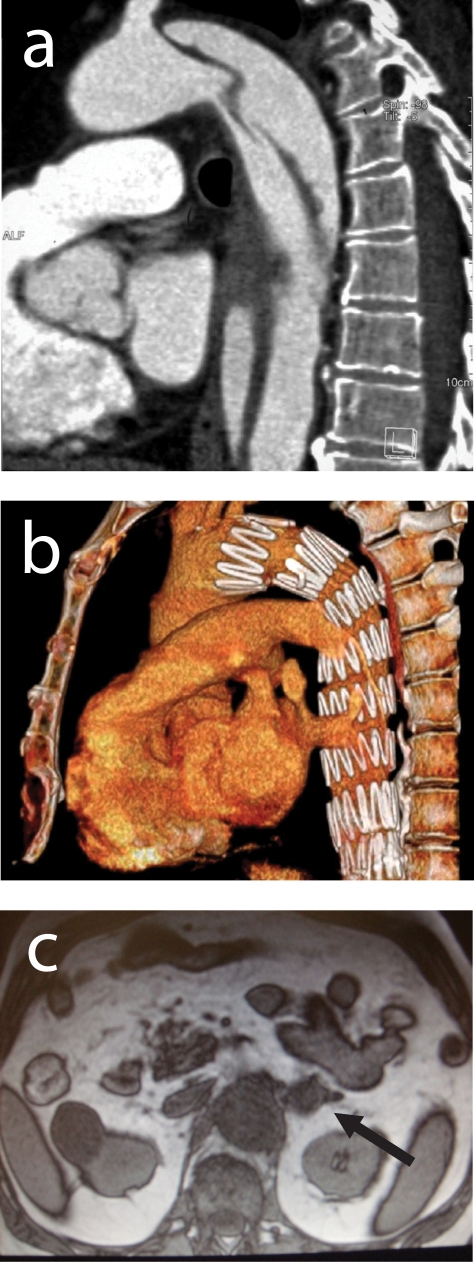
(a) A CT scan of thorax and abdomen demonstrated a dissection starting just below left succlavian artery and extending downward to the left renal artery, involving the celiac tripod and superior mesenteric artery. (b) A postoperative control CT scan demonstrated the correct graft positioning, not endoleaks and the closure of all secondary tears. (c) A MRI of abdomen showing a left adrenal lesion of 2 cm of diameter.

On physical examination the patient was not shocked, his pulse was 87 beats/min, blood pressure 150/105 mmHg. He had truncal obesity, a "moon face" and proximal muscle weakness. An electrocardiogram showed left ventricular hypertrophy. His laboratory work-up revealed anaemia (Hb 10.7 g/dl), hypokaliemia (3.4 mEq/L), metabolic alkalosis (HCO^3^_-_ 35 mmol/L) and hyperglycaemia (170 mg/dl). A CT scan of thorax and abdomen demonstrated a dissection starting just below left succlavian artery and extending downward to the left renal artery, involving the celiac tripod and superior mesenteric artery (Fig 1a). The dissection was classified as Stanford B, De Bakey III. Moreover CT scan of abdomen revealed incidentally a left adrenal tumour of 25 mm of diameter.

The patient was pharmacologically treated and was felt at risk for impending aortic rupture. Therefore, an emergent endograft positioning was planned. Our goal was to exclude the damaged segment of the aorta and to obliterate the entry of blood into the false lumen (both at the initial intimal tear and at the secondary tears along the vessel). Through an inguinal incision, the right common, superficial and profunda femoral artery were dissected free. A prosthetic graft (Valiant, Medtronic, Sunnyvale, CA, USA 42 mm x 198 mm) was placed just below the origin of the left succlavian artery up to the diaphragmatic hiatus. A postoperative control CT scan demonstrated the correct graft positioning, not endoleaks and the closure of all secondary tears (Fig. 1b).

This patient was evaluated for hypercortisolism and the plasma cortisol was increased to 33.9 μg/dl (vn, value of normal, 5.6-23 μg/dl) without circadian rhythm and not suppressed by the administration of 1 mg of dexamethasone (overnight text: 9.8 μg/dl; vn <1.8 μg/dl ). The plasma adrenocorticotropic hormone (ACTH) was less than 10 pg/ml (vn 12-60 pg/ml) and 24h urinary free cortisol excretion (UFC) was elevated 465.6 μg/24 h (vn 26-135 μg/24h ).

A MRI of abdomen was performed to have a more accurate evaluation of the adrenal mass, it was of 2 cm of diameter, well capsulated, hyperintense in T1 sequences with a normal reduction of signal in T2 weighted sequences (Fig 1c).

A diagnosis of CS was made on basis of the endocrinological tests and for the presence of left adrenocortical lesion. Via laparotomic approach, a left adrenalectomy was performed during intravenous replacement of hydrocortisone, ACE-inhibitor, aspirin and insulin therapy.

Six months after adrenalectomy, blood pressure was controlled (135/75 mmHg) with anti-hypertensive therapy, laboratory analysis revealed glycaemia 102 mg/dl, glycated haemoglobin (HbA1c) 5.7%, potassium 4.26 mEq/l, sodium 139.7 mEq/L, plasma creatinine 1.05 g/dl, a.m. plasma cortiso l9.7 μg/dl, plasma ACTH 28 pg/ml and UFC 45 μg/24h.

## Discussion

Primary adrenal hypersecretion of cortisol, mainly from unilateral tumours and more rarely from bilateral adrenal hyperplasia, is responsible for approximately 15-20% of endogenous CS cases. Among the unilateral tumours, adenomas are found in 60% of cases and carcinomas in the remaining 40%. Approximately 10% of primary adrenal CS is due to primary bilateral adrenocortical hyperplasia.

CS is frequently associated with cardiovascular risks (through hypercortisolism), leading to cardiovascular diseases as the mayor cause of death [2]. In particular, the CS is associated with hypertension, impaired glucose metabolism and hyperlipemia, which represent the crucial cardiovascular factors for the atherosclerosis [9, 10].

Studies has shown that unexpected endogenous hypercortisolism occurs in 0.5-1% of patients with hypertension, 2-3% with poorly controlled diabetes, 6-9% with incidental adrenal mass and 11% with osteoporosis and vertebral fractures [11].

Although CS has been known as a risk factor for aorta dissection, the association of these two entities is not common. In our case, at the time of the diagnosis of a Stanford type B aortic dissection, we revealed a CS due to adrenocortical adenoma. To our knowledge, only two case of dissecting aortic aneurysm simultaneously with CS has been found in the English literature [7, 8].

In 1935, Lawrence and Zimmerman [7] described a 44-year-old man who presented with the clinical features of CS of pituitary basophilism and died as the result of a ruptured dissecting aortic aneurysm. In 2004 Takagi et al [8] reported a 55-year-old man who presented CS due to adrenocortical adenoma and a saccular nondissecting true aneurysm of the distal aortic arch with a mural thrombus and a Stanford type B aortic dissection. This patient simultaneously was operated by left adrenalectomy through laparotomy and graft replacement of the distal aortic arch and the proximal descending thoracic aorta under partial cardiopulmonary bypass through a left thoracotomy.

In our patient, a prosthetic graft (Valiant, Medtronic, Sunnyvale, CA, USA 42 mm x 198 mm) was placed firstly just below the origin of the left succlavian artery up to the diaphragmatic hiatus, and later a left adrenalectomy was performed via laparotomic approach.

The mechanisms which lead to dissecting aortic aneurysm in patients with CS remain unclear. A possible explication may be partially attributed to the excess of cortisol secretion with chronic hypercortisolemia which has been demonstrated to cause arteriosclerosis, hypertension and dissecting aneurysm in investigations of experimental model. In fact, some authors have reported that dissecting aortic aneurysm is produced in hamsters by cortisone, and cellular metaplasic transformation of smooth muscle cells to fibroblast-like cells has been observed in the media of the aorta adjacent to aneurysm [12, 13].

One other important factor which should be taken into account is the duration of the disease. In patients with CS there is a positive correlation between duration exposed to the hypercortisolism and the severity of hypertension, which is the most important risk factor for aortic dissection [14].

In conclusion, we presented the third case of patient with Stanford type B aortic dissection and CS caused by adrenocortical adenoma.
